# Insulin-like growth factor binding protein-6 inhibits prostate cancer cell proliferation: implication for anticancer effect of diethylstilbestrol in hormone refractory prostate cancer

**DOI:** 10.1038/sj.bjc.6602520

**Published:** 2005-04-20

**Authors:** H Koike, K Ito, Y Takezawa, T Oyama, H Yamanaka, K Suzuki

**Affiliations:** 1 Department of Urology, Gunma University Graduate School of Medicine, 3-39-22 Showa-machi, Maeabshi, Gunma 3718511, Japan; 2 Department of Tumor Pathology, Gunma University Graduate School of Medicine, 3-39-22 Showa-machi, Maeabshi, Gunma 3718511, Japan

**Keywords:** diethylstilbestrol, IGFBP-6, prostate cancer

## Abstract

Diethylstilbestrol (DES) is a synthetic oestrogen, and its anticancer effects are exerted in androgen-dependent prostate cancer. The administration of DES decreases serum testosterone to castration levels. However, in androgen-independent prostate cancer patients, who are already orchiectomised, the administration of DES improves symptoms and decreases prostate-specific antigen (PSA). The mechanisms responsible for these direct inhibitory effects have been explained as biological actions not mediated by oestrogen receptors. We assessed the gene expression profiles of prostate cancer cells treated with DES, and investigated direct inhibitory effects of DES. DES inhibited the proliferation of LNCaP and PC-3 cells. cDNA microarray analysis showed that expression of many genes was downregulated by DES. However, insulin-like growth factor binding protein 6 (IGFBP-6) gene expression levels were upregulated in PC-3 cells. IGFBP-6 gene expression and protein levels significantly increased after DES treatment. Recombinant IGFBP-6 inhibited cell proliferation, and the inhibitory effect of DES was neutralised by anti-IGFBP-6 antibody. From the immunohistochemical analysis of IGFBP-6 using biopsy samples from androgen-independent prostate cancer, we found IGFBP-6 expression in androgen independent prostate cancer, and that DES treatment increased the IGFBP-6 staining intensity of the cancer cells in one sample. These findings suggested that DES induces IGFBP-6, which inhibits cell proliferation in an androgen-independent prostate cancer cell line, PC-3. IGFBP-6 therefore might be involved in the direct effects of DES in androgen-independent prostate cancer.

Diethylstilbestrol (DES) is a synthetic oestrogen used in the treatment of advanced human prostate cancer (Ferro *et al*, 1989), and diethylstilbestrol diphosphate (DESdP) is a nontoxic prodrug form of DES (Flocks *et al*, 1955; Abramson *et al*, 1982). The palliative effects of DESdP administered to advanced-, hormone-insensitive prostate cancer patients are well known (Flocks *et al*, 1955; Colapinto *et al*, 1961; Band *et al*, 1973; Droz *et al*, 1994; Takezawa *et al*, 2001). DESdP relieves bone pain in the majority of patients with metastatic prostate cancer who receive therapy (Hawtrey *et al*, 1974; Takezawa *et al*, 2001).

The administration of oestrogenic analogues causes the suppression of luteinising hormone-releasing-factor stimulation of the pituitary gland (Paulson, 1984). In addition, the subsequent reduction of testosterone synthesis induces anorchid levels of circulating serum testosterone (Paulson, 1984). The beneficial effect of DES therapy for previously orchiectomised patients, however, indicates that DES may not operate solely through the pituitary–gonadal axis. Several studies have sought to define the mode of DES action on a cellular level. DES treatment inhibits the growth of primary cultures derived from benign hyperplasia and prostate carcinoma samples (Brehmer *et al*, 1972) as well as prostate cancer cell lines (Schulz *et al*, 1990). Intracellular localisation studies of DES inhibition have targeted mitochondrial adenosine triphosphate synthase (McEnery *et al*, 1989), respiratory chain enzymes (Schulz *et al*, 1990), and microtubules (Hartley *et al*, 1985; Yokota *et al*, 1994) as sites of action. Robertson *et al* (1996) reported that DES inhibits proliferation of androgen-dependent and androgen-independent human prostate cancer cell lines by promoting cell cycle arrest, inducing apoptosis through a mechanism not mediated by estrogen receptors. However, the direct effects of DES are unclear. The aim of this study was to investigate the direct effects of DES in terms of gene expression, and to characterise the biological significance of specific genes involved in these effects.

## MATERIALS AND METHODS

### Cell and chemicals

The human prostate cancer cell lines LNCaP and PC-3 were purchased from Dainippon Pharmaceutical (Tokyo, Japan) and cultured in RPMI (Sigma, St Louis, MO, USA) supplemented with 10% fetal calf serum (FCS) (Moregate, Bulimba, Australia). DES (Sigma) was dissolved in DMSO, and recombinant insulin-like growth factor binding protein 6 (IGFBP-6) (Genzyme-techne, Minnesota, USA) was resuspended with PBS and stored at −70°C. Neutralising goat anti-IGFBP-6-antibody (Genzyme-techne), rabbit polyclonal anti-IGFBP-6-antibody (Austral biologicals, CA, USA) and normal goat IgG (Genzyme-techne) were resuspended with water and stored at −70°C, and MTT was purchased from Sigma and dissolved in water at 10 mg ml^−1^.

### Proliferation assay of human prostate cancer cells

Approximately 5 × 10^3^ LNCaP cells per well or 1 × 10^4^ PC-3 cells per well were incubated with 100 *μ*l of culture medium (CM) for 24 h at 37°C in a 5% CO_2_ atmosphere. Thereafter, the CM was aspirated and the cells were incubated with CM containing various concentrations of DES, IGFBP-6, neutralising anti-IGFBP-6-antibody or normal goat IgG followed by a 4 h pulse of 50 *μ*g of MTT (Sigma). Isopropanol with 0.04 N hydrochloric acid (100 *μ*l) was added to lyse the cells. Colour development at a wavelength of 540 nM was measured by a Microplate Readers SOFTmax-J enzyme-linked immumosorbent assay reader (Molecular Devices, Sunnyvale, CA, USA).

### DNA gel electrophoresis

LNCaP and PC-3 cells treated with various concentrations of DES for 72 h were harvested by trypsin treatment. Genomic DNA was extracted using Wizard DNA extraction kit (Promega, Madison, WI, USA). DNA pellets were resuspended in distilled water and loaded in a 1.0% agarose gel. DNA was electrophoresed for 1.5 h at 10 V cm^−1^. Gels were stained with ethidium bromide (Sigma) and visualised under UV light.

### cDNA microarray analysis of the gene expression profiles of LNCaP and PC-3 cells

cDNA microarray analysis of the gene expression profiles of LNCaP and PC-3 cells were performed according to previously described method (Suzuki *et al*, 2002). The cells were cultured in CM with 50 *μ*M DES for 48 h, after which total RNA was extracted using an RNeasy Kit (Qiagen, Valencia, CA, USA) and treated with DNase I (Invitrogen, Carlsbad, CA, USA). In total, 50 *μ*g of DNase I treated total RNA was reverse-transcribed in the presence of 35 *μ*M Cy3-dUTP or Cy5-dUTP (Amersham Pharmacia, Piscataway, NJ, USA) and 10 × lowT dNTP mix (5 mM dATP, 5 mM dGTP, 5 mM dCTP, 2 mM dTTP) using an RNA Fluorescence Labelling Core Kit (Takara, Tokyo, Japan). In total, 100 pg of *λ* polyA+RNA (Takara) was added as a positive control. Unincorporated nucleotides and salts were removed by chromatography with a Centrisep (Princeton Separations, Adelphia, JN, USA). The Cy3 or Cy5 labelled cDNA after purification was mixed, and 30 *μ*g of human Cot I DNA (Invitrogen), 16 *μ*g of poly-dA (Amersham Phamacia) and 20 *μ*g of yeast tRNA (Sigma) were added to reduce nonspecific binding. The probes were resuspended in 10 *μ*l of hybridisation solution consisting of 6 × SSC, 0.2% SDS and 5 × Denhardt's solution (Invitrogen), and incubated at 98°C for 2 min and then 4°C for 10 s. An IntelliGene™ Human Cancer CHIP Version 2.1 (Takara) glass slide was treated with prehybridisation solution consisting of 6 × SSC, 0.2% SDS, 5 × Denhardt's solution and 1 mg ml^−1^ denatured salmon sperm DNA (Invitrogen). The denatured probe was placed onto the glass slide with a coverslip, and hybridisation was performed at 65°C for 15 h. The slides were then washed twice for 30 min in 2 × SSC at 55°C, 5 min in 2 × SSC at 65°C and 5 min in 0.05 × SSC at room temperature.

The fluorescence intensities of the immobilised targets were measured using an Affymetrix Array Scanner 418 (Affymetrix, Santa Clara, CA, USA). The two fluorescent images (Cy3 and Cy5) were separately scanned and the resulting imaging files were saved. The fluorescence intensities were analysed using the imaging software ImaGene™ ver 3.0 (BioDiscovery, Los Angeles, CA, USA). IntelliGene™ Human Cancer CHIP ver 2.1 (Takara) carries 557 genes related to cancer (URL: http://www.takara.co.jp/bio/go ods/new/new6/new6-7.htm) and contains the following control spots: housekeeping genes: beta-actin; Uba80 mRNA for ubiquitin; phospholipase A2; glyceraldehydes-3-phosphate dehydrogenase (GAPDH); tubulin, alpha2; major histocompatibility complex, class I, A; ribosomal protein S5; general transcripton factor IIB; hexokinase 1 F0; DNA from a non-human species: lambda-A, lambda-B, lambda-C, lambda-D, lambda-E, pUC19, E.coli ompA, E.coli ftsZ, *Arabidopsis* chlorophyll *ab* binding protein. The normalisation constant from 76 spots of 12 housekeeping genes was used to calculate the calibrated ratio for every cDNA spot within the image. We then calculated the differential expression ratios from two independent experiments and omitted spots for which the fluorescence intensities of Cy3 and Cy5 were less than 2000.

### Quantification of mRNA levels

Quantification of transcript levels was performed using a Light Cycler (Roche Diagnostics,Indianapolis, IN, USA) according to the manufacturer's protocol and previous reports (ICJ, ACR). LNCaP cells were cultured in CM with 50 *μ*M DES, and PC-3 cells were culutured in CM with 12.5–50 *μ*M DES for 48 h. Total RNA was then extracted as described above and 2 *μ*g of this was reverse transcribed using random primers (Invitrogen), according to the manufacturer's protocol. Amplification was performed in 18 *μ*l of QuantiTect SYBR Green PCR Master MIX (Qiagen) using 2 *μ*l of cDNA and forward and reverse primers. Next, PCR was performed for one cycle of 15 min at 95°C followed by 50 cycles of 15 s at 94°C, 20 s at 60°C and 15 s at 72°C, with fluorescence detection being carried out at 72°C after each cycle. After the final cycle, melting-point analysis of all samples and controls was performed from 65 to 95°C. Absolute quantification of IGFBP-6 gene levels was performed according to a previously described method (Suzuki *et al*, 2002). For standard curve generation, PCR products of target genes (known amounts) were used for template DNA, and for the internal control 18s ribosomal RNA (rRNA) transcript levels were used. Gene expression levels were expressed as fold changes of those of the controls. The sequences of the primers for IGFBP-6 and 18s rRNA were as follows: IGFBP-6: forward, 5′-AGG ATG TGA ACC GCA GAG AC-3′, reverse, 5′-GGT AGA AGC CTC GAT GGT CA-3′; 18 s rRNA: forward, 5′-CGG CTA CCA CAT CCA AGG AA-3′, reverse, 5′-GCT GGA ATT ACC GCG GCT GC-3′.

### Detection of IGFBP-6 protein by Western blotting

Detection of IGFBP-6 protein was performed according to modification of a previously described method (Hasumi *et al*, 2003). Equal amounts of proteins (100 *μ*g) from PC-3 cells were electrophoresed on a 5–20% SDS–polyacrylamide gel and transferred onto a nitrocellulose membrane. After blocking, the membrane was reacted with anti-IGFBP-6 antibody at a 1 : 500 dilution at room temperature for 1 h. The membrane was then reacted with horseradish peroxidase-linked rabbit immunogloburin, and recombinant IGFBP-6 was visualised using an ECL detection system (Amersham Biosciences, Tokyo, Japan). Data were obtained from three experiments.

### Immunohistochemical analysis of IGFBP-6 in clinical tissues

To investigate the direct effects of DESdP, we examined androgen-independent prostate cancer which initially responded favourably to primary hormonal therapy, but then showed increases in prostate-specific antigen (PSA) levels or clinical symptoms or new lesions during treatment (Takezawa *et al*, 2001). Diethylstilbestrol diphosphate (DESdP) at 250 mg day^−1^ was dissolved in 500 ml of physiological saline and intravenously infused for 28 days. PSA levels were measured before and 28 days after DESdP administration was started by enzyme immunoassay (EIA) (TOSOH-II PA kit, Tosoh Tokyo, Japan). Before and after DESdP administration, a biopsy of the prostate was performed. Paraffin sections of 4-*μ*m thickness were cut from archival paraffin blocks containing representative histology of the lesion. An immunohistochemical study was then performed on the sections using the avidin-biotinylated peroxidase complex (ABC) method with rabbit polyclonal anti-IGFBP6 antibody (kindly provided by Dr J Martin, University of Sydney, St Leonards, Australia) at 1 : 2000 dilution. Briefly, paraffin sections were dewaxed with xylene and incubated with 0.3% hydrogen peroxide in absolute methanol to block endogenous peroxidase activity. Antigen-retrieval was then carried out by autoclave-heating for 5 min. To avoid nonspecific staining, the sections were incubated with 10% normal goat serum for 30 min at room temperature and were then treated with primary antibodies at 4°C overnight. After a thorough washing, the sections were incubated with the secondary biotinylated anti-rabbit IgG (Vector Laboratories, CA, USA) for 30 min at room temperature followed by the ABC complex (DakoCytomation Co. Ltd., Glostrup, Denmark) for 30 min at room temperature. Finally, the slides were visualised with a solution containing 0.02% 3,3′-diaminobenzidine tetrahydrochloride and 0.005% H_2_O_2_, followed by light counterstaining with haematoxylin.

## RESULTS

**Figure 1:**
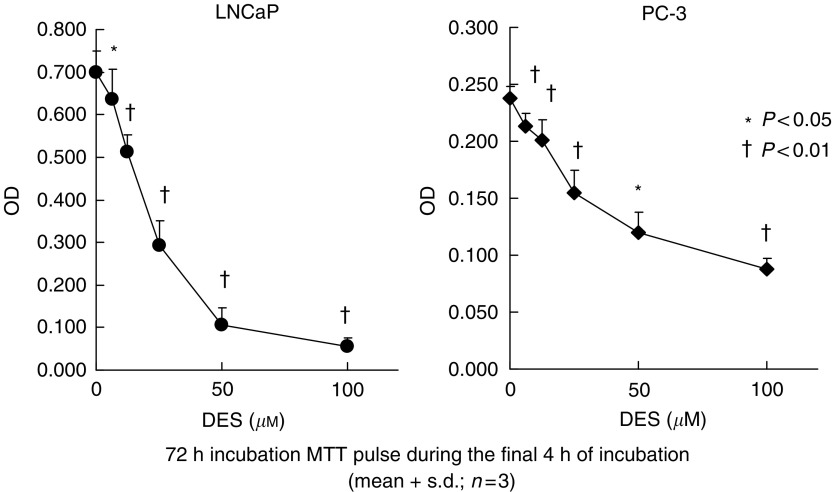
Inhibition of the proliferation of human prostate cancer cell lines LNCaP and PC-3 by diethylstilbestrol (DES). Cells were incubated for 24 h, and thereafter CM was aspirated away and the cells were incubated with CM containing various concentrations of DES. After 72 h, the number of viable cells was measured by MTT assay. Optical densities (OD) of cell lysates were measured at a wavelength of 540 nm. The values are expressed as means+s.d. (*n*=3), and the *P*-values were <0.05 (^*^) and <0.01 (†).

**Figure 2:**
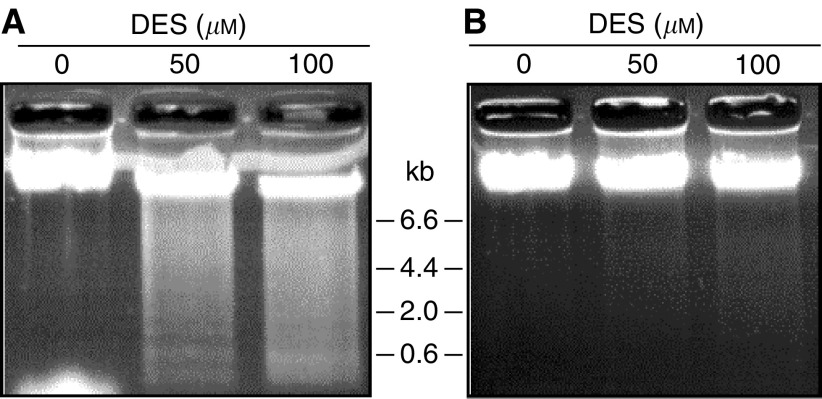
Gel electrophoresis of DNA of LNCaP and PC-3 treated with diethylstilbestrol (DES). Cells treated with various concentrations of DES for 72 h were harvested, and DNA was extracted. DNA was electrophoresed in a 1.0% agarose gel. DNA fragmentation was observed after DES treatment in both LNCaP (**A**) and PC-3 cells (**B**).

DES induces apoptosis in prostate cancer cells, and so we first performed a cell proliferation assay using MTT to examine the effect of DES on prostate cancer cell proliferation. Viable cell numbers of LNCaP and PC-3 cells were decreased after DES administration in a dose-dependent manner as shown in Figure 1. To confirm the occurrence of apoptosis, extracted genomic DNA from LNCaP and PC-3 cells were electrophoresed. As shown in Figure 2, DNA fragmentation was observed in both cells after DES treatment; however, the extent of DNA fragmentation was low in PC-3 cells in comparison with that in LNCaP cells.

To screen for the effect of DES on prostate cancer cells, changes in gene expression in LNCaP and PC-3 cells was assessed using a cDNA microarray. Two experiments showed that more than half of the genes fixed on the slide glass were downregulated greater than two-fold, and genes that were downregulated greater than five-fold are shown in Table 1. Genes involved in cell attachment/invasion, cell cycle, intracellular signalling, apoptosis and cell proliferation are included. On the other hand, only four genes were upregulated greater than two-fold after DES treatment as shown in Table 2. IGFBP-6 and keratin 19 gene expression levels were upregulated only in androgen-independent PC-3 cells. In clinical practice, DES exerted its antitumour effect on androgen-independent prostate cancer (Takezawa *et al*, 2001). Furthermore, IGFBP-6 inhibited the proliferation of rhabdomyosarcoma cells (Gallicchio *et al*, 2001). These findings prompted us to study the effect of DES and IGFBP-6 on androgen-independent prostate cancer PC-3 cells.

**Figure 3:**
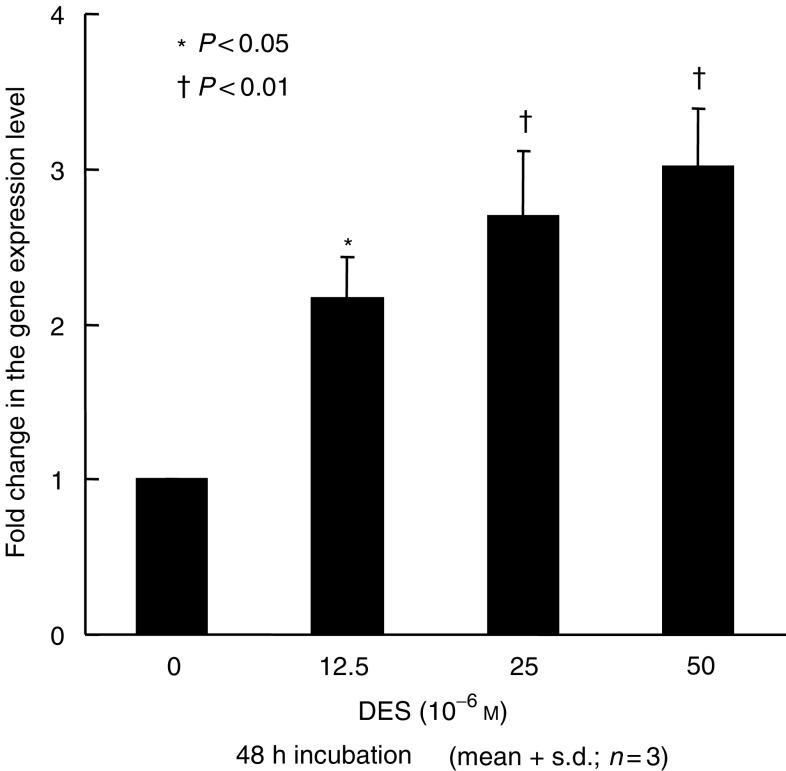
Insulin-like growth factor binding protein-6 (IGFBP-6) gene expression levels in PC-3 cells. Transcript levels were measured by quantitative real-time PCR after treatment with various concentrations of DES. 18s ribosomal RNA transcript levels were used for the internal control, and gene expression levels were expressed as fold changes relative to those of the controls. IGFBP-6 gene expression was significantly increased after DES treatment in a dose-dependent manner in PC-3 cells. The values are expressed as means+s.d. (*n*=3), and the *P*-values were <0.05 (^*^) and <0.01 (†).

**Figure 4:**
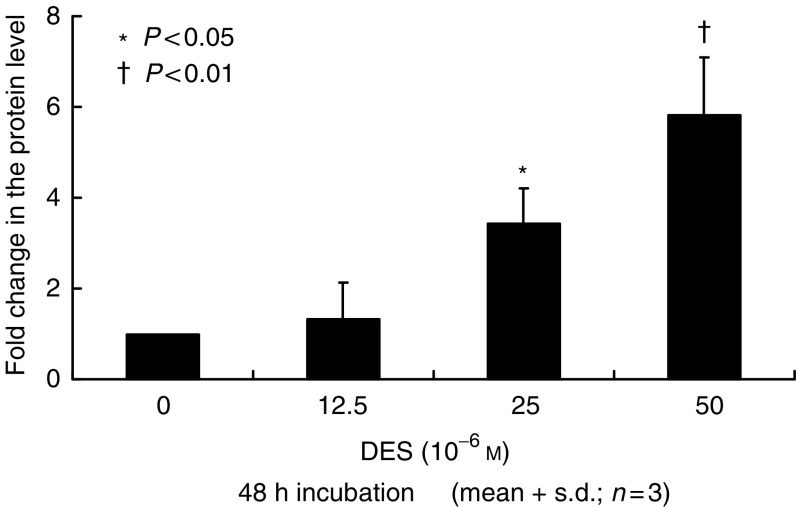
Insulin-like growth factor binding protein-6 (IGFBP-6) protein levels in PC-3 cells. Protein levels were measured by Western blot analysis after treatment with various concentrations of DES. Proteins levels were expressed as fold changes. IGFBP-6 protein expression was significantly increased after DES treatment in a dose-dependent manner in PC-3 cells. The values were expressed as means+s.d. (*n*=3), and the *P*-values were <0.05 (^*^) and <0.01 (†).

To confirm the gene expression levels of IGFBP-6 in LNCaP and PC-3 cells, quantitative real-time PCR was performed. Basal expression levels of IGFBP-6 in LNCaP cells were about 20-fold lower than those in PC-3 (data not shown). After incubation with 50 *μ*M DES for 48 h, IGFBP-6 gene expression levels in LNCaP cells showed no significant differences in comparison with the controls (data not shown). In PC-3 cells, IGFBP-6 gene expression levels significantly increased after DES treatment in a dose-dependent manner as shown in Figure 3. Western blot analysis showed that IGFBP-6 protein levels significantly increased in a dose-dependent manner as shown in Figure 4.

**Figure 5:**
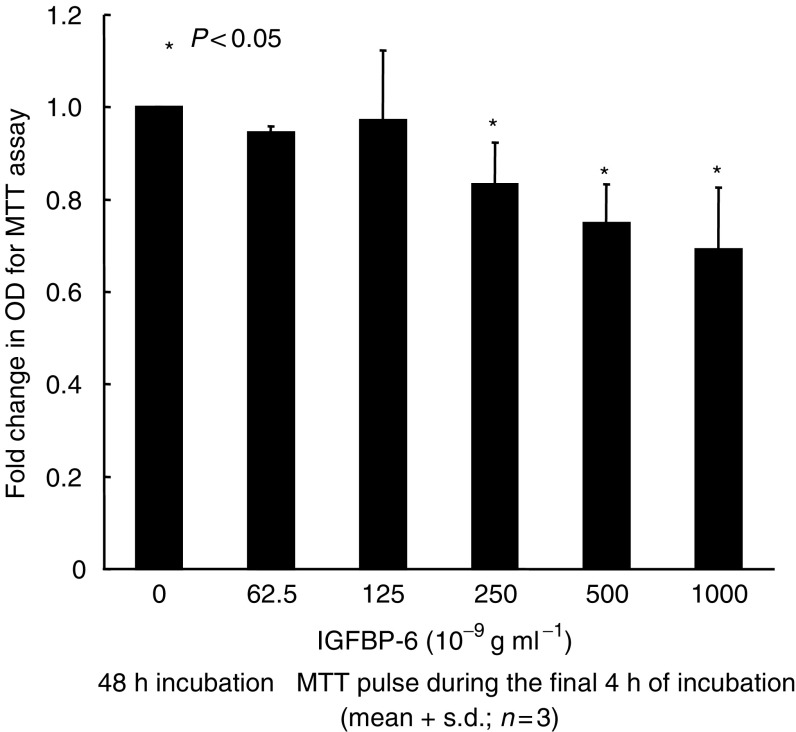
Inhibition of proliferation of human prostate cancer PC-3 cells by recombinant IGFBP-6. Cells were incubated for 24 h, and thereafter CM was aspirated away and the cells were incubated with CM containing various concentrations of IGFBP-6. After 48 h, the viable cell number was measured by MTT assay. The optical density (OD) of the cell lysates is expressed as the fold change. Recombinant IGFBP-6 inhibited the proliferation of PC-3 cells in a dose-dependent manner. The values are expressed as means+s.d. (*n*=3), and the *P*-values were <0.05 (^*^).

**Figure 6:**
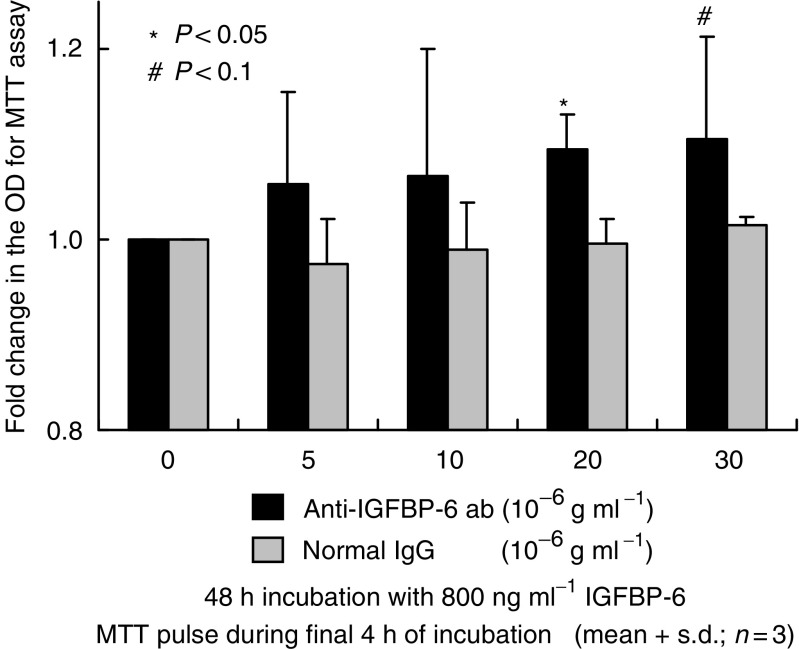
Effects of IGFBP-6 and anti-IGFBP-6 antibody on PC-3 cell proliferation. Cells were incubated for 24 h, and thereafter CM was aspirated away. After 48 h of incubation of PC-3 cells with 800 ng ml^−1^ recombinant IGFBP-6 and various concentrations of anti-IGFBP-6 or normal goat IgG, the viable cell number was measured by MTT assay. The optical density (OD) of the cell lysates was expressed as the fold change. In total, 20–30 *μ*g ml^−1^ of anti-IGFBP-6 antibody significantly neutralised the inhibitory effects of cell proliferation. The values are expressed as means+s.d. (*n*=3), and the *P*-values were <0.05 (^*^) and <0.1 (#).

**Figure 7:**
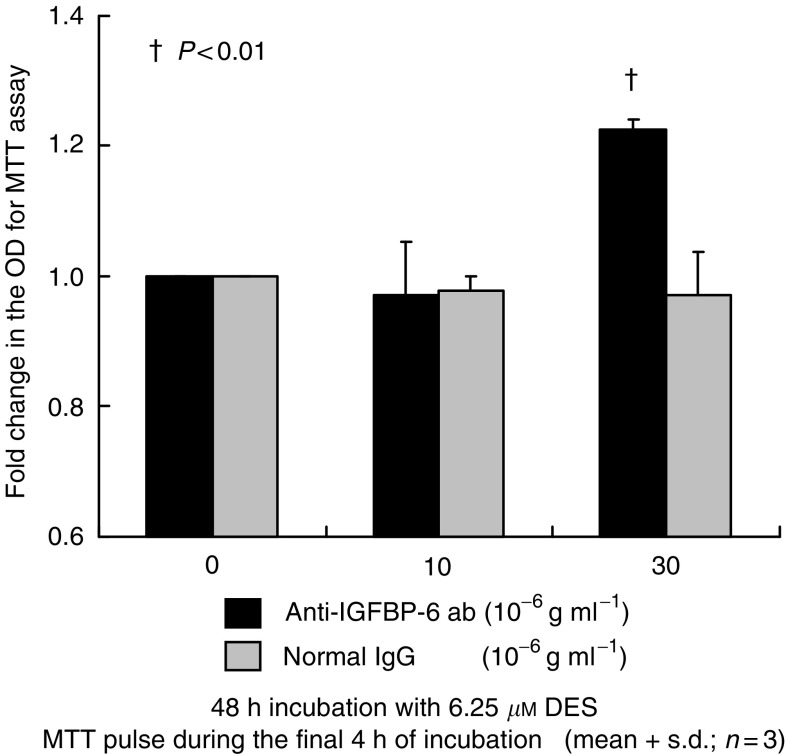
Effect of neutralising anti-IGFBP-6 antibody on DES-treated PC-3 cells. Cells were incubated for 24 h, and thereafter CM was aspirated away. After 48 h incubation of PC-3 cells with 6.25 *μ*M DES and various concentrations of anti-IGFBP-6 antibody or normal goat IgG, the viable cell number was measured by MTT assay. The optical density (OD) of the cell lysates was expressed as the fold change. Cell proliferation was significantly upregulated by incubation with high-dose antibody (30 *μ*g ml^−1^). Anti-IGFBP-6 antibody inhibited DES-induced cell growth suppression. The values are expressed as means+s.d. (*n*=3), and the *P*-values were <0.01 (†).

We next studied the effect of recombinant IGFBP-6 on prostate cancer cells. Although low-dose IGFBP-6 (62.5–125 ng ml^−1^) did not affect cell proliferation, high-dose IGFBP-6 (250–1000 ng ml^−1^) inhibited cell proliferation as shown in Figure 5. After incubating the PC-3 cells with 800 ng ml^−1^ recombinant IGFBP-6, 20–30 *μ*g ml^−1^ anti-IGFBP-6 antibody neutralised the inhibitory effects of cell proliferation. However, 5–10 *μ*g ml^−1^ anti-IGFBP-6 antibody did not significantly neutralise this effect (Figure 6). Finally, we examined the effect of neutralising anti-IGFBP-6 antibody on DES treated PC-3 cells. Low-dose antibody (10 *μ*g ml^−1^) did not show a significant effect on cell proliferation, but, cell proliferation was significantly upregulated with incubation with high-dose antibody (30 *μ*g ml^−1^) as shown in Figure 7. Normal goat IgG did not affect on cell proliferation, as shown in Figures 6 and 7.

**Figure 8:**
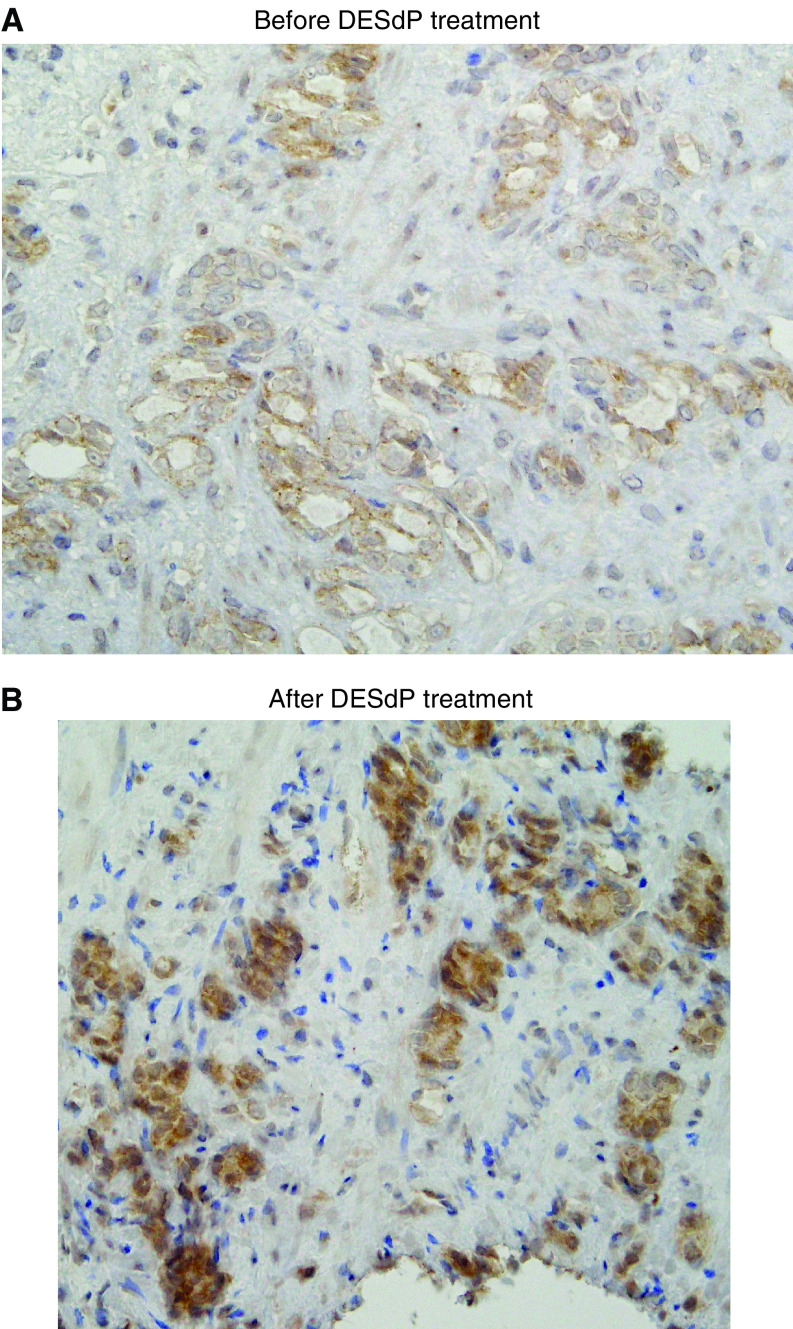
Immunohistochemical staining of IGFBP-6. IGFBP-6 was visible in the cytoplasm of cancer cells in the organ (**A**). For one patient, DESdP treatment increased the IGFBP-6 staining intensity in cancer cells (**B**). The immunostained section in (**B**) is from the same patient as that shown in (**A**). Cells that stained positively for IGFBP-6 are *brown* in colour.

Clinically, DESdP treatment decreased PSA levels in all patients (Takezawa *et al*, 2001). In the prostate biopsy tissues before and after DES-dP treatment, cancer cells were detected in four patients. IGFBP-6 expression was examined by immunohistochemical staining, and was visible in the cytoplasm of cancer cells in the organ. Before treatment with DESdP, IGFBP-6 expression was positive in three patients. In one of them (patient #1), IGFBP-6 expression increased after treatment with DESdP, and in others (patients #2 and #7) it was not changed (Table 3). DESdP treatment increased the IGFBP-6 staining intensity in cancer cells of patient #1 as shown in Figure 8.

## DISCUSSION

We first confirmed the antiproliferative and apoptosis-inducing effects of DES on prostate cancer cells. Cell proliferation of LNCaP or PC-3 cells was inhibited by DES in a dose-dependent manner in combination with cellular apoptosis. This is consistent with results reported by Robertson *et al* (1996), who examined the induction of apoptosis by DES in PC-3 cells. They concluded that the direct cytotoxic effects of DES in prostate cancer cells are estrogen receptor-independent and involve the promotion of cell cycle arrest and apoptosis.

Recently, gene expression profiles can be screened using a cDNA microarray, which provides important information on biological activities. We previously reported the gene expression profiles of LNCaP and PC-3 cells treated by genistein using a cDNA microarray, and found unique genes involved in the direct effects of genistein on prostate cancer cells (Suzuki *et al*, 2002). The present study showed that expression levels were downregulated for most genes. This downregulation reflects the suppressive effects of DES. However, the expression of IGFBP-6 and keratin 19 gene were upregulated in androgen-independent prostate cancer PC-3 cells. IGFBP-6 inhibits the proliferation of rhabdomyosarcoma cell lines (Gallicchio *et al*, 2001). Therefore, we characterised the biological action of IGFBP-6 in PC-3 cells. In rhabdomyosarcoma cells, IGFBP-6 inhibited cell proliferation in a dose-dependent manner by inhibiting insulin-like growth factor II (IGF-II), which stimulates cell proliferation. Gallicchio *et al* concluded that IGFBP-6 exerts an inhibitory effect on the proliferation and survival of rhabdomyosarcoma cells *in vitro*. In the present study, the proliferation of PC-3 cells was inhibited by recombinant IGFBP-6 in a dose-dependent manner. We also found that DES administration induces IGFBP-6 at the mRNA and protein level. Furthermore, anti-IGFBP-6 antibody antagonises the inhibitory effect of DES. Therefore, we speculate that IGFBP-6 is involved in the direct effect of DES.

IGFBP-6 is unique for its dramatically higher affinity for IGF-II (Bach *et al*, 1999), and is an inhibitor of IGF-II (Kiefer *et al*, 1992; Bach *et al*, 1994; Bach *et al*, 1999). IGF-II is an autocrine growth factor that affects neuroblastoma (Grellier *et al*, 2002) and rhabdomyosarcoma (Gallicchio *et al*, 2001) cells. *In vivo*, Grellier *et al* reported on the transplantation of human IGFBP-6-expressing neuroblastoma cells in nude mice, and their results showed a lower incidence of xenografts, which also exhibited slower growth than those obtained using control cells (1998). IGF-II was more strongly expressed in control tumours in this model. They concluded that excess IGFBP-6 displaces IGF-II from IGFBP-2, thus preventing it from potentiating the mitogenic action of IGF-II (2002). Gallicchio *et al* (2001) also reported that IGFBP-6 dramatically inhibits xenograft growth of rhabdomyosarcoma cells.

In prostate cancer, Kimura reported that IGF-II occurred at the protein and RNA level in PC-3 cells without the occurrence of significant amounts of IGF-I protein in the conditioned media of these cell-lines. Furthermore, IGF-II stimulates the growth of PC-3 cells. Therefore, the autocrine activity of IGF-II may contribute in part to the proliferation of PC-3 cells (Kimura *et al*, 1996). Drivdahl reported that the upregulation of IGFBP-6 occurs in association with the activity of 1,25-dihydroxyvitamin D_3_ in prostate cancer cells, and suggested a role for IGFBP-6 in the suppression of prostate tumour cell growth (Drivdahl *et al*, 1995). This prompted us to investigate the effect of IGFBP-6 on androgen-independent prostate cancer PC-3 cells. The present findings are the first observations to confirm the inhibitory effect of IGFBP-6 on PC-3 cells.

In clinical practice, we treated seven patients with androgen-independent prostate cancer with DESdP (Table 3). PSA levels decreased in all patients, and palliative effects were obtained. Viable cancer cells were confirmed by prostate needle biopsy specimens for four patients. Three of the four were positive for IGFBP-6 staining. For one patient, IGFBP-6 immunostaining was upregulated as shown in Figure 8. This suggests that upregulation of IGFBP-6 was observed in a part of patients with androgen-independent prostate cancer treated by DESdP. We considered that the heterogeneity of biological features of prostate cancer may have affected the present results.

In conclusion, we found that many genes including cell attachment/invasion, cell cycle, intracellular signalling, apoptosis and cell proliferation genes, are downregulated after DES treatment in human prostate cancer LNCaP and PC-3 cells. However, IGFBP-6 is upregulated at the mRNA and protein level in androgen-independent human prostate cancer PC-3 cells. rIGFBP-6 inhibits PC-3 cell proliferation, and neutralising anti-IGFBP-6 antibody antagonises the inhibitory effect of DES in PC-3 cells. These finding suggest that IGFBP-6 might be involved in the direct effect of DES in androgen-independent human prostate cancer.

## Figures and Tables

**Table 1:** Gene List: reduced after DES treatment

**Category**	**GenBank Acc. No.**	**Gene name**	**LNCaP**	**PC-3**
Cell attachment/cell invasion	U41766	A disintegrin and metalloproteinase domain 9	•	•
	Z13009	Cadherin 1 (E-cadherin)	•	
	D21255	Cadherin 11 (osteoblast)		•
	X87838	Catenin, beta 1	•	•
	M14648	Integrin, alpha V	•	
				
Cell cycle	X05360	Cell division cycle 2	•	•
	S72008	Cell division cycle 10	•	•
	X51688	Cyclin A2	•	
	M74091	Cyclin C	•	•
	X77784	Cyclin G1	•	•
	U47414	Cyclin G2	•	•
	X17644	G1 to G2 phase transition 1	•	•
				
Intracellular signalling	AL035071	MAP kinase 1	•	•
	X80692	MAP kinase 6	•	•
	L35263	MAP kinase 14	•	•
	L11284	MAPK kinase 1	•	•
	M97935	STAT-1, 91 kDa	•	•
				
Apoptosis	U37547	Apoptosis inhibitor 1	•	•
	NM012103	Apoptosis inhibitor 2	•	•
	NM013979	BCL2/adenovirus E1B 19 kDa interacting protein 3	•	•
	U13737	Caspase 3	•	•
				
Others	J04088	Topoisomerase (DNA) II, alpha	•	•
	M15796	PCNA	•	

• indicates that the gene expression level was reduced more than five-fold after DES treatment.

**Table 2:** Gene list: elevated after DES treatment

**GenBank Acc. No.**	**Gene name**	**LNCaP**	**PC-3**
D86962	Growth factor receptor-bound protein 10	•	
X57351	IFN induced transmembrane protein 2	•	
NM002178	Insulin like growth factor binding protein-6		•
Y00503	Keratin 19		•

• indicates that the gene expression level was elevated more than two-fold after DES treatment.

**Table 3:** Clinical and immunohistchemical data of androgen-independent prostate cancer patients

		**Immunohistochemical analysis of IGFBP6 in prostate biopsy tissues**		**PSA (ng dl^−1^)**	
**Patient**	**Pathology at diagnosis**	**Before DES-dP treatment**	**After DES-dP treatment**	**Before**	**After**
1	Poorly diff.	Positive	More positive	821	238
2	Moderately diff.	Positive	Positive	417	194
3	Moderately diff.	No cancer cells	No cancer cells	25.3	9.3
4	Moderately diff.	No cancer cells	No cancer cells	166	112
5	Poorly diff.	No cancer cells	No cancer cells	35.6	2.4
6	Poorly diff.	Negative	Negative	641	261
7	Poorly diff.	Positive	Positive	305	23.6
